# Vanishing industries and the rising monopoly of universities in published research

**DOI:** 10.1371/journal.pone.0202120

**Published:** 2018-08-14

**Authors:** Vincent Larivière, Benoit Macaluso, Philippe Mongeon, Kyle Siler, Cassidy R. Sugimoto

**Affiliations:** 1 École de bibliothéconomie et des sciences de l’information, Université de Montréal, Montréal, QC, Canada; 2 Observatoire des Sciences et des Technologies, Centre Interuniversitaire de Recherche sur la Science et la Technologie, Université du Québec à Montréal, Montréal, QC, Canada; 3 Centre for Science and Technology Studies, Leiden University, Leiden, The Netherlands; 4 Copernicus Institute of Sustainable Development, Utrecht University, Utrecht, The Netherlands; 5 School of Informatics and Computing, Indiana University, Bloomington, IN, United States of America; Institut Català de Paleoecologia Humana i Evolució Social (IPHES), SPAIN

## Abstract

Anecdotes abound regarding the decline of basic research in industrial and governmental settings, but very little empirical evidence exists about the phenomenon. This article provides a systematic and historical analysis of the contribution of various institutional sectors to knowledge production at the world and country levels across the past four decades. It highlights a dramatic decline in the diffusion of basic research by industrial and governmental sectors across all countries—with a corresponding increase in the share from universities—as well as an increase of partnerships between universities and other sectors. Results also shows an increase in the relative share of industries in applied research, as measured through patents. Such divergence in university and industry research activities may hinder industries’ ability to translate basic knowledge into technological innovation, and could lead to a growing misalignment between doctoral training and future job expectations. Industries and universities must rethink strategies for partnerships and publishing to maximize scientific progress and to ensure the greatest gains for society.

## Introduction

Bell Labs was once one of the most prominent industrial research laboratories in the United States—associated with eight Nobel prizes and lauded for major technological developments such as the transistor and the laser. However, personnel declined from 30,000 to 1,000 employees between 2001 and 2009, as a result of the termination of its basic research program [1,2]. Basic science saw similar disinvestment in Canada a few years later when the century-old National Research Council (NRC)—Canada’s largest research organization, associated with three of the country’s Nobel prizes—explicitly changed funding priorities to deemphasize basic research in favor of applied work that would appeal to the industrial sector [3]. These types of scientific policy changes and philosophies can be linked to structural changes in industrial research and development (R&D) characterized by the outsourcing of basic research, downsizing of in-house basic research activities, and prioritization of firms’ core competencies [4]. In short, for firms thinking about the immediate commercial viability of products and for scientific benefactors who desire immediate, concrete results, basic research can seem like an uncertain and risky endeavor.

Conversely, universities have become active participants in applied research. In the United States, the founding and expansion of business and engineering schools in the mid-20^th^ Century created new links between universities and industries, while also creating professional homes for many research scientists who had formerly been employed in industry [5]. The passing of the Bayh-Dole act in 1980 propelled innovation in universities, by allowing academic institutions to pursue and retain profits from patenting activities. Similar policies have diffused throughout the developed world, underpinning extensive research and commercial activity in universities [6]. Through patenting and educating a skilled workforce, universities serve as a major source of economic development and activity in the contemporary economy [7]. However, with declining research funds available in many universities, some academic researchers have turned to industry funding as a means for conducting research, often with productive and mutually beneficial outcomes [8]. Such perceived decline of industrial research contrasts with the thesis of Gibbons et al. [9] who, more than two decades ago, heralded the demise of the academic control of science—and the corresponding rise of other sectors’ research activities. While several studies have since sought to demonstrate the central role of universities, as well as the contribution of other sectors to the production of knowledge [10,11,12,13,14,15], they have failed to provide historical or global trends. This paper addresses this gap by providing a historical analysis of institutional sectors’ basic and applied research production across the past four decades, using scholarly articles and utility patents as evidence of this production.

## Materials and methods

### Data sources

This paper uses two data sources: the Web of Science for the 1980–2014 period (N = 28,420,363 papers) and the United States Patent and Trademark Office (USPTO) database for the 1976–2014 period (N = 4,969,210). Both databases have been transformed into SQL relational databases to allow for the compilation of advanced bibliometric indicators.

### Assignment of institutional sectors in papers

Articles, notes, and reviews published between 1980 and 2014 and indexed in Thomson Reuters’ Web of Science containing affiliation data were included in the analysis. These papers contained 58,015,879 institutional addresses, which were coded into four sectors (universities, governments, industry, and other), following common practice [10,15] using a set of keywords (Appendix 1). The institutional field of WoS was mined for evidence of these keywords. Hospitals—generally marked in WoS with “HOSP”—were either categorized in the universities sector when they contained the string “UNIV”—as in UNIV-HOSP—, in the industrial sector when they had markers such as “INC” or “CORP”, or in the others sector when they could not be categorized in the two previously mentioned categories.

While this method does not lead to a high assignation of distinct institutions (Table 1)—with only 57.7% of institutions having a sector attributed—these institutions are responsible for the large majority of papers. Indeed, this assignation process has led to the attribution of a sector for 95.9% of fractionalized papers (obtained by calculating institution and sector’s share of each paper, and then summing it for all papers), and for 94.8% of paper-institutions combinations (Table 1). The proportion of fractionalized papers to which a sector could be assigned varied by country: for example, while only a small proportion of fractionalized papers for the United States (2.1%), Japan (1.9%), China (2.1%), Canada (2.0%) could not be assigned a sector, this percentage is higher for Germany (5.8%), UK (4.9%), France (4.3%), as well as for USSR/Russia (16.2%).

**Table 1:** Sectoral assignation of fractionalized number of papers, of paper-institution combinations and of distinct institutions.

Sector	Fractionalized N. Papers		Paper-Institution Combinations		Institutions	
			N	%	N	%
Governments	2,251,488	7.9%	4,010,115	9.8%	178,583	8.8%
Industries	1,401,617	4.9%	2,370,779	5.8%	346,509	17.0%
Universities	20,241,316	70.9%	26,872,555	65.5%	331,452	16.3%
Others	3,476,657	12.2%	5,639,372	13.8%	318,585	15.6%
Unknown	1,168,082	4.1%	2,116,279	5.2%	862,583	42.3%
All Sectors	28,539,160	100.0%	41,009,100	100.0%	2,037,712	100.0%

### Publication indicators

Results are presented as fractionalized numbers of papers, to take into account the increase in interinstitutional collaboration. For instance, a paper that has three universities, one industry, and one government address will count as 0.6 of a university, 0.2 of an industry, and 0.2 of a government paper. These fractionalized numbers of articles are then summed for each sector and divided by the total number of articles, to obtain the relative contribution of each sectors’ to publication activity at the world and country levels. For this calculation, unknown sectors are removed from the denominator. Citation indicators are field-normalized. We compile, for each speciality and each publication year, the percentile rank of each paper in the citation distribution, and aggregate for each sector the proportion of papers that fall in the top 5% most cited. When the share of top 5% most cited papers is above 5%, it means that the sector scores above average; when it is below, it means the opposite. Disciplines and specialities used are those of the U.S. National Science Foundation in its Science and Engineering Indicators series, which are based on the journals in which researchers publish. These are grouped into five broad domains to assess sector’s contribution to world papers.

### Assignment of institutional sectors in patents

In order to compare publication patterns across sectors, we assigned institutional sectors to assignees (i.e., owners of the commercialization rights to a patent) of utility patents granted by the USPTO over the 1976–2014 period (N = 4,969,210). Patents contain two categories of assignees: individuals and organisations. Hence, only institutional assignees were assigned a sector; patents owned by individuals were assigned in the “individuals” sector. For institutional assignees, the same sectoral assignation key and procedure described above for papers was used in patents, with a few adaptations. For instance, while institutions names in WoS are abbreviated (UNIV for University, “GOV” for governments, etc., see: https://images.webofknowledge.com/WOK46P9/help/WOS/h_adabrv.html), they appear in full in the USPTO database. Hence, we use full strings rather than abbreviated ones.

As shown in Table 2, the share of unknown distinct institutional assignees (11.0%) is much lower than that obtained for publications. However, for fractionalized number of patents and of patent-assignees combinations, the proportion of unknowns is quite similar to that obtained for scholarly papers. As one might expect, most institutional assignees of patents are industries, representing 84.7% of all assignees, 89.9% of patent-assignees pairs, and 90.3% of fractionalized numbers of patents.

**Table 2:** Sectoral assignation of fractionalized numbers of patents, of patent-assignees combinations and of distinct assignees.

Sector	Fractionalized N. Patents		Patent-Assignees Combinations		Assignees	
			N	%	N	%
Governments	57,099	1.3%	59,359	1.3%	8,088	0.8%
Industries	3,953,185	90.3%	3,968,809	89.8%	849,549	84.7%
Universities	115,089	2.6%	123,338	2.8%	28,353	2.8%
Others	11,327	0.3%	12,470	0.3%	4,741	0.5%
Unknown	243,468	5.6%	254,400	5.8%	109,859	11.0%
All Sectors*	4,380,168	100.0%	4,418,376	100.0%	1,003,115	100.0%
Individuals	732,155	-	734,929	-	-	-

* excluding individuals

### Patent indicators

While co-ownership of patents (i.e., two or more assignees on the same patent) is much less frequent than interinstitutional collaboration papers, we nonetheless compiled sectors’ share of patent assignees as a fractionalized numbers of patents. For instance, a patent with one industry and one individual as assignees would could as 0.5 of an industrial patent and 0.5 of an individual patent. These fractionalized numbers of patents are then summed for each sector and divided by the total number of patent, to obtain the relative contribution of each sectors’ to patenting activities at the world levels. For this calculation, unknown sectors are removed from the denominator.

## Results

**Fig 1:**
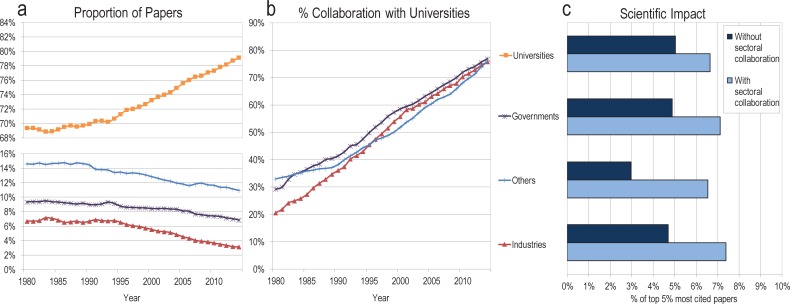
(A) Proportion of fractionalized papers, (B) proportion of papers written in collaboration with universities, and (C) scientific impact, by sector (world level), 1980–2014.

Fig 1 presents the share of papers published by each institutional sector, as well as collaborative and scientific impact trends. It shows that there has been a marked increase in the share of papers published by universities at the world level, with corresponding decreases in all other sectors (Fig 1A). This trend is observed for most of the most productive countries (Figs A and B in S1 File), but is particularly marked in the US, where the number of fractionalized papers from industry decreased from more than 15,500 in the beginning of the nineties to less than 9,900 in 2014. The dominance of universities in the dissemination of research is even more apparent when considering collaboration: for example, the number of industry papers co-authored with universities increased from 21% in 1980 to 76% in 2014 (Fig 1B). A similar trend can be observed across all other sectors, demonstrating that non-university sectors are not only less active in producing research papers, but increasingly collaborative with universities when they publish. These collaborative relationships have mutual benefits: papers co-authored with researchers affiliated with other sectors are more cited than single-sector publications (Fig 1C).

**Fig 2:**
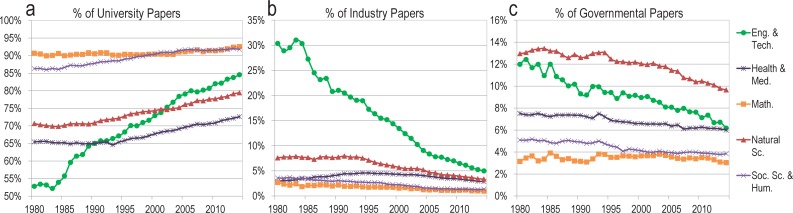
**Proportion of world papers authored by(A) Universities, (B) Industries and (C) Governments, by discipline, 1980–2014**.

There are strong disciplinary variations in the portfolio of published research by sector (Fig 2). Over the last three decades, universities have maintained an almost complete monopoly over publications in mathematics, social sciences, and humanities (Fig 2A). In contrast, while the university share of engineering and technology publications was less than 60% in the 1980s, it has increased to 87% in 2014—a consequence of the decline in the share of industrial research in this area (from 25% to 3%) (Fig 2B). Governments are most active in the natural sciences (Fig 2C), which can be traced to historical governmental mandates in areas like wildlife management, environment, and natural resources. However, the share of governmental papers declined by about 33% in natural sciences, health, and medicine, and by about 50% in engineering and technology between 1980 and 2014.

**Fig 3:**
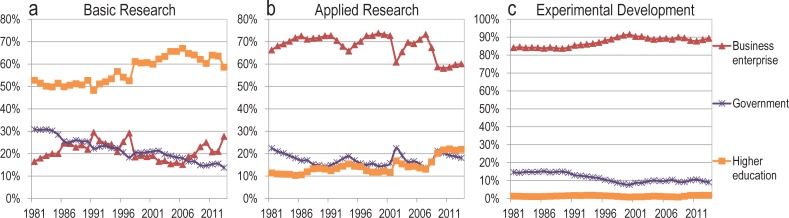
**Funding of (A) basic research, (B) applied research and (C) experimental development, by sector of execution, United States, 1981–2013 (OECD Statistics)**.

**Fig 4:**
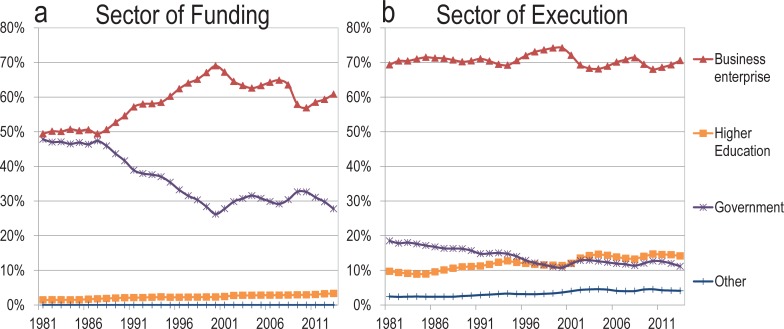
**Proportion of (A) funding and (B) execution of R&D activities, by sector, United States, 1981–2013 (OECD Statistics)**.

Our results highlight a dramatic decline in the diffusion of research by the industrial and governmental sectors, with a parallel increase in the share of partnerships between academe and other sectors. While for the United States these results are consistent with OECD data on the *production* of basic research (Figs 3 and 4) performed by governments—which declined from 31% in 1981 to 14% in 2013—they may appear inconsistent with OECD data for industries showing relatively stable *investments* of industry in basic research [13]. That is, the OECD confirms a decrease in basic research activity in the governmental sector—mostly to the benefit of the higher education sector—, but an increase in investments in basic research in industries over the last 10 years, as well as a stable proportion of all types of executed R&D throughout the period. This discontinuity suggests at least two possible explanations: either published research is poorly related to what the OECD categorizes as basic research and industries are indeed decreasing their basic research activities; or industries perform basic research at the same (or increasing) rate but have declined proportionally—and absolutely in some countries like the US and the UK—to *disseminate* this work in published research. In short, industries are either adopting a model of secrecy or disseminating research in other ways.

**Fig 5:**
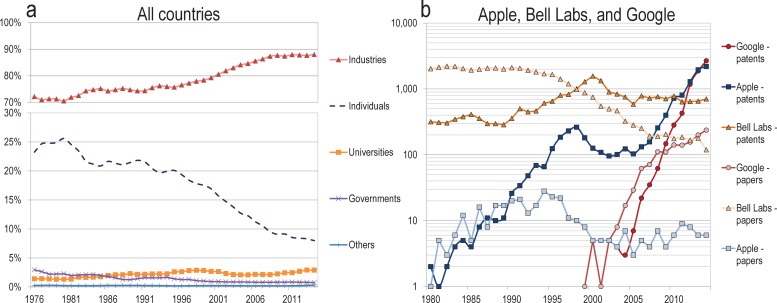
(a) Share of patent ownership (USPTO) by institutional sector, all countries, 1976–2014, (b) number of papers published and patents owned, Apple, Bell Laboratories (and various owners) and Google, 1980–2014.

Scholarly papers are not the only evidence of an active research enterprise. Patenting is another source of evidence for research activity. Therefore, to understand the state of research by sector one must also account for this mode of production. An examination of historical patenting trends (Fig 5) demonstrates the increasing dominance of industry in patenting, increasing from 73.7% in 1976 to 87.6% in 2014, mainly at the expense of patents owned by individual inventors. This suggests that industries remain actively involved in research—at least, in the type of research that leads to patenting—but does not demonstrate whether this happens at the expense of research production. To test this, we compiled historical patenting and publication rates for three well-known private firms with established research portfolios: Bell Laboratories, Apple, and Google. All three cases show a decline of basic research production and an increase in patenting activity. Bell Laboratories registered 5 times more papers than patents for most of the 1980s, but this ratio was reversed in 2014. Apple registered a similar number of papers and patents until the 1990s. However, the company has not published more than 10 papers in a given year since 1998, but registered 2,200 patents in 2014. Google also published more papers than it filed patents until 2009, but registered 10 times more patents than published papers in 2014. These cases are exemplary, but suggest a shifting focus from papers to patents in the industrial sector.

## Discussion and conclusion

Universities are moving towards a nearly exclusive monopoly over the publication of scientific articles. Industries, on the other hand, are vanishing from this production model, despite an infusion of investments in basic research and being increasingly dominant the patent production space. This suggests that there is not a decline in industries’ research activity, but rather in its diffusion to the scientific community. This secrecy of basic research in industry—and corresponding concentration of publication of basic research in universities—threatens both the pursuit of basic knowledge and industrial competitiveness. For instance, it may lead to a gradual depletion of the collective pool of basic knowledge [16], which is essential to maintain innovative capacity and translate basic knowledge into technological innovation [17]. The type of research pursued by industry can complement purely academic pursuits: science in industry has been shown to be less bound by theoretical matters and more prone to speculation, which can be conducive to innovation [18]. Evidence of the complementary nature of these sectors is shown in the citation advantage for collaboration: partnerships between university and industry realizes the highest scientific impact. This has also been demonstrated in the success of geographic research clusters, such as Silicon Valley, Route 128, and the North Carolina Research Triangle. Our results suggest that a diversity of actors in the scientific system leads to the highest impact results.

All sectors are relying more heavily on collaborations with universities for publications. However, the bridge between universities and industry is routinely crossed by university-trained doctoral students [19–21]. This may lead to several misalignments, due to various value systems in academe and industry [22]: While university researchers are increasingly rushing to “publish or perish”, there is no such culture in industries and governments, where other forms of knowledge diffusions are privileged (e.g., patents in industry and white papers and reports for governmental research). In other words, while incentives for academics to publish have increased, no such incentives exist for firms. In contrast, industrial authors may be reticent to publish for fear of divulging trade secrets and losing competitive advantage [23]; a hypothesis that is reinforced by the growing emphasis of the industrial sector on patenting. This may explain why, despite accounting for a stable share of R&D expenditure—both in terms of funding and execution (Fig 4)—and for an increasing proportion of basic research expenditures (Fig 3), firms publish less, both in absolute and relative terms. It may not be surprising, therefore, that doctoral students seeking industrial positions place a lower value on publishing than their university-oriented peers [24]. Despite this, firms with higher proportions of young, university-trained researchers produce more scientific publications [25], perhaps a holdover from their training.

As case studies of European and Japanese firms have shown [26], firms publish when performing long term research and to signal the existence of tacit knowledge in their organizations. Thus, decline in industrial publishing activities suggest that these functions are becoming less important. Disclosure in industry has also been problematized by issues of competition and intellectual property, despite advances in open innovation [27]. While there may be industrial advantages to “selective” [28] and “free” revealing [29], our results reinforce that an open science strategy [30] is neither the norm nor the direction of industries. Given that open flows and interchanges of knowledge and labor between institutions is conducive to innovation and success in industry [31], institutional norms and strategies that promote excessive secrecy can be myopic and could undermine industry-wide productivity, and at odd with an increasingly open scientific environment [32,33].

An open flow of ideas and information among sectors is essential to remain competitive in the contemporary knowledge economy. The growing monopolization of published research by universities and the vanishing of industries from this communicative space represents a major threat to this economy. Unidirectional flows of information have negative effects upon all sectors for knowledge production and innovation. Industries and universities must rethink strategies for partnerships and publishing to maximize scientific progress and to ensure the greatest gains for society.

## Supporting information

S1 File List of character strings for assignation of sectors and three supplementary figures. (DOCX)
