# Supplementary material for: Vanishing industries and the rising monopoly of universities in published research
Source: PLoS One. 2018 Aug 14;13(8):e0202120. doi: 10.1371/journal.pone.0202120 (PMC6091964; doi:10.1371/journal.pone.0202120)
Supplement: S1 File — (DOCX) [file pone.0202120.s001.docx]

**Supplementary material**

**List of character strings for assignation of sectors**

**Universities**

"UNIV"

"UNIVERSIDAD"

"UNIVERSITAT"

"UNIVERSITE"

"UNIVERSITY"

"INTERUNIV"

"FAK"

"COLL"

"FAC"

"CUNY"

"CALTECH"

"MIT"

"IIT"

"NYU"

"NTH"

"SCH"

"ECOLE"

"UER"

"U"

"FAK"

"UNIVERSITAIRE"

"HSCH"

"SUNY"

FINDSTRING(Institution,"INST-TECHNOL",1) > 1) AND NOT ‘HOSP’

**Industries**

"AB"

"ABB"

"ABC"

"ABP"

"AE"

"AG"

"ALCATEL"

"ALCOA"

"AS"

"ASSOCIATES"

"AY"

"BASF"

"BL"

"BT"

"BV"

"BVBA"

"CBS"

"CCC"

"CORP"

"CV"

"CVBA"

"CYF"

"DUPONT"

"EBVBA"

"EC"

"EE"

"EEIG"

"EHF"

"EPE"

"EURL"

"EV"

"FCP"

"FIRMA"

"FKF"

"FMA"

"GBR"

"GE"

"GEC"

"GK"

"GMBH"

"GMBH"

"GMK"

"GSK"

"HB"

"HF"

"IBM"

"IKS"

"KB"

"KF"

"KFT"

"KG"

"KGAA"

"KHT"

"KK"

"KKT"

"KV"

"KY"

"LDA"

"LLC"

"LLLP"

"LLP"

"LP"

"LTD"

"MAATSCHAP"

"MEPE"

"NBC"

"NL"

"NUF"

"NV"

"NYRT"

"OE"

"OHG"

"OY"

"OYJ"

"PARTG"

"PLC"

"PLLC"

"PP"

"PTY"

"RCA"

"RHF"

"SARL"

"SAS"

"SASU"

"SCA"

"SCS"

"SF"

"SGPS"

"SICAF"

"SICAV"

"SK&F"

"SKA"

"SKF"

"SL"

"SLL"

"SLNE"

"SNC"

"SPAREBANK"

"SPJ"

"SPK"

"SPP"

"SPZOO"

"SRA"

"SRL"

"STICHTING"

"TEO"

"TMI"

"VOF"

"YK"

"ZRT"

(word == "INC" && order_word != 1)

(word == "ANS" && order_word != 1)

(word == "APS" && order_word != 1)

(word == "ASA" && order_word != 1)

(word == "CO" && order_word != 1)

(word == "CPT" && order_word != 1)

(word == "DA" && order_word != 1)

(word == "IS" && order_word != 1)

(word == "SA" && order_word != 1)

(word == "SAPA" && order_word != 1)

(word == "SPA" && order_word != 1)

FINDSTRING(Institution,"ICI-PLC",1) > 0

FINDSTRING(Institution,"UNILEVER",1) > 0

**Governments**

"NIA"

"NIDDKD"

"INFM"

"CEA"

"BNL"

"ANL"

"AERE"

"OFFICE"

"BOARD"

"AFRC"

"GVT"

"AGCY"

"BUR"

"CDC"

"CDCP"

"CEN"

"CERN"

"CENS"

"CENFAR"

"CNEN"

"CNR"

"CSIC"

"CSIRO"

"CNRS"

"DESY"

"DFVLR"

"DSIR"

"EPA"

"EURATOM"

"FAA"

"FAO"

"FCC"

"FTC"

"GOVT"

"INRA"

"INSERM"

"MAFF"

"MINIST"

"NASA"

"NCI"

"NEI"

"NHLBI"

"NIAID"

"NIAMDD"

"NICHHD"

"NIDR"

"NIH"

"NIMH"

"NINCDS"

"NOAA"

"ORNL"

"ORSTOM"

"RAF"

"UKAEA"

"USDA"

"USAF"

"FDA"

"EPA"

"USN"

"NIEHS"

Institution == "US-DOE"

Institution == "AFL-CIO"

Institution == "US-PHS"

Institution == "US-DEPT"

Institution == "FIC"

Institution == "NCI"

Institution == "NCCAM"

Institution == "NCMHD"

Institution == "NCRR"

Institution == "NEI"

Institution == "NHLBI"

Institution == "NHGRI"

Institution == "NIA"

Institution == "NIAAA"

Institution == "NIAID"

Institution == "NIAMS"

Institution == "NIBIB"

Institution == "NICHD"

Institution == "NIDCD"

Institution == "NIDCR"

Institution == "NIDDK"

Institution == "NIDA"

Institution == "NIEHS"

Institution == "NIGMS"

Institution == "NIMH"

Institution == "NINDS"

Institution == "NINR"

Institution == "NLM"

Institution == "CDC"

Institution == "ATSDR"

Institution == "EPO"

Institution == "NCBDDD"

Institution == "NCCDPHP"

Institution == "NCEH"

Institution == "NCHS"

Institution == "NCHSTP"

Institution == "NCID"

Institution == "NCIPC"

Institution == "NIOSH"

Institution == "NIP"

Institution == "PHPPO"

Institution == "US-FOREST-SERV"

Institution == "US-GEOL-SURVEY"

FINDSTRING(Institution,"NATL-LAB",1) > 0

FINDSTRING(Institution,"RES-COUNCIL",1) > 0

FINDSTRING(Institution,"KOREA-INST-SCI-&-TECHNOL",1) > 0

**Hospitals**

"NHS"

"HOSP"

"CLIN"

"HOP"

"KLIN"

"CHU"

"CHR"

FINDSTRING(Institution,"OSPED",1) > 0

FINDSTRING(Institution,"MED-CTR",1) > 0

**Others**

"CEGEP"

"PLANCK"

"WHO"

"IEEE"

"IEE"

"IAE"

"UNICEF"

"UNESCO"

"ASTM"

"ASME"

"MUSEUM"

"EG"

"CVOA"

"AMBA"

"PARTNERSHIP"

"PC"

"OK"

"ANL"

"STIFTELSE"

"SCOP"

"BA"

"VERENGING"

"SPOLDZIENLNIA"

"CRL"

"EK"

"KOOPERATIV"

"ASSOC"

Institution == "SERC"

Institution == "MRC"

FINDSTRING(Institution,"RES-FDN",1) > 0

**Supplementary tables and figures**

Figure S1. Share of research output by institutional sector, top 20 most active countries, 1980-2014

Figure S2. Share of research output (log scale) by institutional sector, top 20 most active countries, 1980-2014

Figure S3. Number of papers by sector and discipline, 1980-2014
